# Heart failure myocardial perfusion swine study with semi-quantitative analysis

**DOI:** 10.1186/1532-429X-13-S1-P50

**Published:** 2011-02-02

**Authors:** Ting Song, Maureen N Hood, Jeffrey A Stainsby, Vincent B Ho

**Affiliations:** 1 GEHC ASL, Bethesda, MD, USA; 2 USUHS&NNMC, Bethesda, MD, USA; 3 GEHC ASL, Toronto, ON, Canada

## Introduction

Cardiac perfusion imaging is a recognized method for non-invasive evaluation for myocardial ischemia. However, it is unclear how global heart failure affects myocardial perfusion. In this study, we explore semi-quantitative perfusion in a Yorkshire swine heart failure study.

## Methods

In this study, five Yorkshire swine were implanted with pacemakers with the intent of producing tachycardia-induced heart failure. Each pig was scanned on a 1.5T MR scanner (GE Medical System, Waukesha, WI, USA) at baseline and at time of heart failure. For MR acquisition, we used a standard fast gradient echo perfusion acquisition with an 8-channel cardiac coil, 40 phases, 128x128, 7mm slice, 2RR, and with free breathing. 0.2mmol/kg dose of Gadoteridol was injected intravenously at 1ml/sec. Signal intensity (SI) time courses were evaluated for the myocardium and the blood pool. Arrival time, peak time, slope, maximum upslope, and contrast enhancement ratio (CER) were calculated using Cine Tool (GE Medical System). Index is defined as the value of myocardium weighted by the value of the blood pool. In addition, semi-quantitative analysis of slope index, maximum upslope index, and CER index were calculated. A paired t-test was evaluated in each measured parameter group to identify the statistical difference between two scans.

## Results

**Figure 1:**
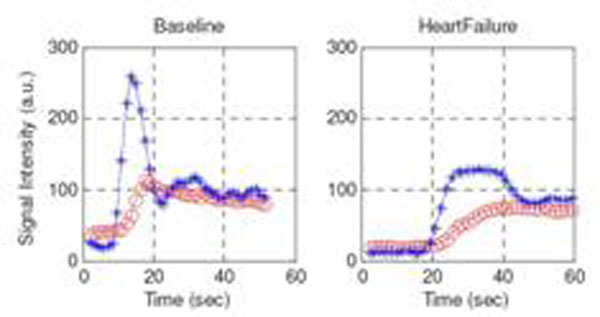
Example signal intensity curves of blood pool (start: *) and myocardial perfusion response (circle: o) are shown in base line (left) and heart failure (right).

**Figure 2:**
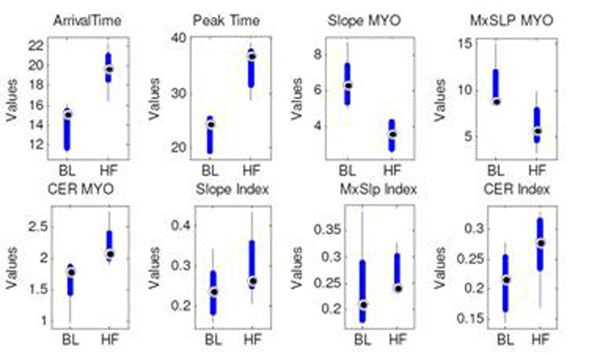
Box plot of the baseline and heart failure groups: arrival time, peak time, slope, maximum upslope, CER, slop index, maximum upslope index, and CER index from left to right. The box has lines at median (circle dot), the lower and upper quartile values (wider box bar end). Whiskers extend from each end of the box to the adjacent values in the data.

Average left ventricular ejection fraction at baseline was 45% ± 4%, and at heart failure was 16% ± 7%. Example signal intensity curves are shown in Figure 1. The signal intensity (SI) curves of baseline myocardial perfusion were sharper and narrower than those at heart failure. In comparison, the myocardial perfusion curves at heart failure were slower and wider. The semi-quantitative parameter results are listed in Table 1. Statistically significant differences were seen in arrival time, peak time, and the slope only. The box plot of the data is shown in Figure 2.

**Table 1:** Parameters with mean and standard deviation values from perfusion analysis

	Arrival Time (sec)	Peak Time (sec)	Slope (a.u.)	MxSlope (a.u.)	CER (a.u.)	Slope Index (a.u.)	MxSlope Index (a.u.)	CER Index (a.u.)
Baseline	13.9±2.2	22.7±3.2	6.5±1.4	10.4±2.8	1.7±0.4	0.24±0.07	0.24±0.09	0.21±0.05
Heart Failure	19.6±2.1	34.9±4.2	3.4±0.8	6.3±2.5	2.2±0.3	0.30±0.09	0.27±0.04	0.27±0.06
p-Value	0.012	0.016	0.025	0.068	0.067	0.330	0.624	0.246

## Discussion

There is currently limited report of myocardial perfusion applied in heart failure. In this study of tachycardia-induced heart failure in swine, perfusion shows delayed arrival time, delayed peak time, and reduced slope of enhancement in myocardium. Future work is needed to explore the potential role of myocardial perfusion imaging applied to heart failure patients, as the ability to identify coexistent myocardial ischemia may be problematic in this population.

